# Evaluation of Ultrasound Measurement of Subcutaneous Fat Thickness in Dairy Jennies during the Periparturient Period

**DOI:** 10.3390/ani12111359

**Published:** 2022-05-26

**Authors:** Irene Nocera, Francesca Bonelli, Luca Turini, Alessio Madrigali, Benedetta Aliboni, Micaela Sgorbini

**Affiliations:** 1 Institute of Life Sciences, Sant’Anna School of Advanced Studies, Via Santa Cecilia 3, 56127 Pisa, Italy; 2 Department of Veterinary Sciences, University of Pisa, Viale delle Piagge 2, 56122 Pisa, Italy; francesca.bonelli@unipi.it (F.B.); alessio.madrigali@phd.unipi.it (A.M.); benedetta.aliboni@libero.it (B.A.); micaela.sgorbini@unipi.it (M.S.); 3 Department of Agriculture, Food and Environment, University of Pisa, Via del Borghetto 80, 56124 Pisa, Italy; luca.turini@agr.unipi.it

**Keywords:** jennies, real time ultrasonography, subcutaneous fat, periparturient period

## Abstract

**Simple Summary:**

Ultrasonography is an accurate method to objectively measure subcutaneous fat (SF) thickness and to routinely predict body fat reserves in cows, horses, and donkeys. The aim of the present study was to describe ultrasonographic SF thickness in periparturient jennies. The SF was evaluated in 6 different truncal location in 6 dairy jennies prior and post parturition. Measurement values were reported and each site were compared through time. No statistically significant differences were found between sites and time. The mean values of SF measurements were above those reported by others. A good and reliable association was detected between body condition score (BCS) and sites during the whole study period. Our study gives a preliminary description of different body locations fat reserves evaluated by ultrasonography, showing no significative variations, in pregnant and lactating jennies.

**Abstract:**

The body condition score (BCS) represents a practical but subjective method for assessing body fat reserves. Real time ultrasonography (RTU) has been proposed as an accurate method to objectively measure subcutaneous fat (SF) thickness and predict body fat reserves in cows, horses and donkeys. The aim of the present study was to describe RTU measures of SF thickness during periparturient period in jennies. The present prospective cohort study evaluated six dairy jennies. SF RTU were performed at 15 and 7 days before the presumptive delivery, and 2, 15 and 30 days after delivery. A portable ultrasound machine and multifrequency linear transducer (5–7.5 MHz) was used. RTU images were obtained in six sites (S1–S6). Results at each time point were reported as mean ± standard deviation and compared through time. A total of 180 images were evaluated. RTU technique was easy to perform and well tolerated. No statistically significant differences were found of each site during time, except for S2 and S6a: S2 at T2 and S6a at T1 were significatively different to values obtained at T5. The RTU mean values were above those reported by others, suggesting major physio-logical challenges related to energy balance and fat mobilization in pregnant jennies bred for milking production. BCS and sites through observational time have shown a good and reliable association. Our study could give preliminary indications on fat reserves in different body locations evaluated thanks to RTU and it show no significative variation of SF thickness, in pregnant and lactating jennies.

## 1. Introduction

Donkeys (*Equus asinus*) have been close companions of humans worldwide and they have been used for several purposes, such as working animals, for milk and meat production or in animal-assisted therapy [1]. Among these, donkey farming is becoming more popular due to donkey milk being used as an alternative to cow’s milk for children’s intolerances and in the cosmetic industry due to its organoleptic properties [2,3]. With the increasing interest in donkeys as dairy animals, studies concerning their health and welfare have been raised [4,5,6,7,8].

Metabolic problems leading to mobilization of body energy reserve influence significantly dairy animal productivity, health and welfare. The transition period represents a challenging physiological stage for dairy animals, due to the increasing energy demand related to milk production and to the decrease in feed intake, which results in the mobilization of lipids from body fat reserve [3]. Milking jennies may present similar metabolic diseases to cows, ewes and goats [9,10]. Thus, also in this species it is essential to assess and monitor body energy reserve in the late pregnancy and ready after foaling. The body condition score (BCS) represents a practical method for assessing body fat reserves, however, BCS evaluation is based on subjective assessment of visual appraisal and palpation of some anatomical landmarks, which could lead to inaccurate results [11]. Moreover, in donkeys, evaluation of fatty neck score has been proposed as a subjective assessment that has been shown to positively correlate to BCS and affect dorsal profile measurements in body condition assessment [12,13]. Real-time ultrasonography (RTU) has been proposed as an accurate method to objectively measure subcutaneous fat (SF) thickness to routinely predict body fat reserves in cows [14,15]. The use of RTU in equids has been closely investigated, both in horses [16,17] and donkeys [11,17,18]. RTU gives a good tissue resolution, is cheap, is well accepted by owners and animals and is easy to perform on manually restricted animals under field conditions [17].

To the best of the authors’ knowledge, there are no studies evaluating the use of RTU in pregnant and lactating jennies. Thus, the aim of the present study was to describe RTU measures of SF thickness during the periparturient period in jennies, from 2 weeks before the delivery to 1 month after.

## 2. Materials and Methods

### 2.1. Animals

The present prospective cohort study was conducted during the foaling season of 2019, following the approval by the Institutional Animal Care and Use Committee of the University of Pisa (OPBA, Pisa, prot. n. 22/19). An owner’s written consent was obtained.

Six dairy jennies belonging to the regional stud farm ‘Le Bandite di Scarlino’ (Grosseto, Italy) and were housed at the Veterinary Teaching Hospital (VTH) ‘Mario Modenato’, Department of Veterinary Sciences, University of Pisa (Italy) were included. All jennies belonged to the Amiata donkey breed, the height at withers ranged between 145 cm and 120 cm (median value, 135 cm), and the body weight at the beginning of the study ranged between 280 and 370 kg (median value, 345 kg), and they were aged between 7 to 11-year-old and equally manage. They were pluriparous and the average parity number was 4.62 ± 2.34. At the regional stud farm, ‘Le Bandite di Scarlino’ jennies were housed in a collective outdoor paddock (100 × 100 m) provided with a shelter. They were fed standard hay *ad libitum* and supplemented individually with 2 kg of commercial equine fodder (Progeo, Masone, Italy), in line with the nutrient requirements stated by the NRC recommendations [19]. All the animals were routinely vaccinated for Equine Influenza and Tetanus and dewormed every 6 months with 200 µg/kg ivermectin *per* kg of body weight (BW). Reproduction activity is through natural breeding and the pregnancy check was carried out within one month from the presumed mating.

Since the last 2 months of pregnancy, all the jennies were moved to the VTH to monitor foaling. The diet of the animals did not change. At the VTH, jennies were housed in collective outdoor paddocks (3 animals/each), where they got used to the new place. Before 15 days of the expected parturition, jennies were transferred into delivery boxes (6 × 6 m) and monitored via a closed-circuit television system. Jennies were kept with their own donkey foals until 15 days after parturition.

### 2.2. Inclusion Criteria

To be included in the study, jennies had to deliver normally, without obstetric assistance and must be healthy based on complete physical examination through the whole study period. Parturition was defined as “normal” if jennies were delivered in recumbency, if the allantochorion rupture and fetal expulsion were unassisted, if the fetus presentation was dorsal anterior, if the second stage of foaling length ≤ 20 min, and if natural rupture of the umbilical cord and placental expulsion occurred within 120 min after parturition [6,20].

### 2.3. Study Design

Body weight was registered at admission time for each jenny, and it was measured using an appropriate scale for equids. Ultrasonography measurements of the SF thickness and BCS evaluation were performed in the study population at 15 (T1) and 7 days (T2) before the presumptive parturition, and 2 (T3), 15 (T4) and 30 days (T5) after delivery. BCS was evaluated by visual appraisal and palpation, according to 1–9 points scale [21]. Contextually, a complete physical examination was performed to assess the health status of the included animals [22].

### 2.4. RTU Assessment of SF Thickness

RTU examinations were performed by holding the jenny in its own box, without any chemical restrain. RTU was performed using a real-time B-mode with a portable ultrasound machine (MyLab30Gold, Esaote, Florence, Italy) with a multifrequency linear transducer (5–7.5 MHz). Machine setting was made following literature recommendations [11] using a frequency of 7.5 MHz, 82% gain, and 6 cm of depth. The hair was clipped and shaved before the examination and alcohol coupled with US gel were applied to provide appropriate contact [11], at each time (T1–T5).

**Figure 1:**
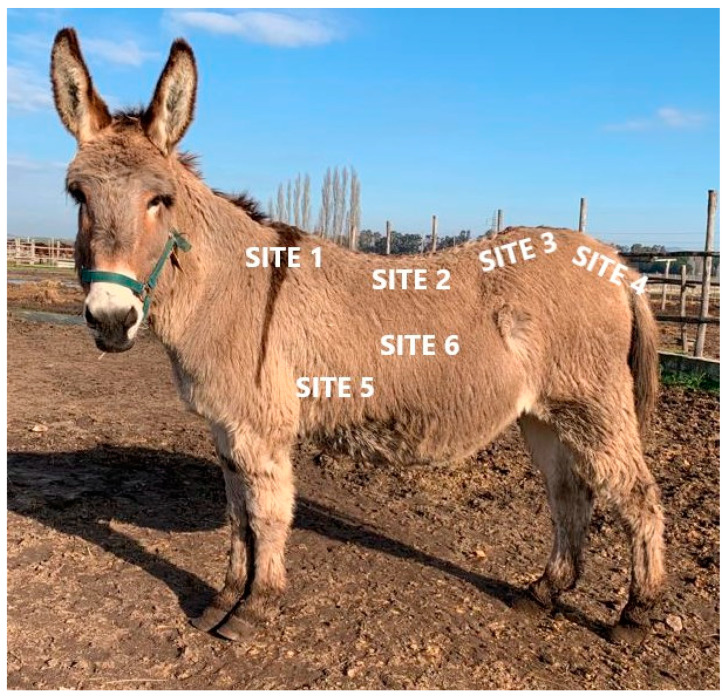
Visual representation of probe placement for RTU site images. At S1 and S2 the probe was placed perpendicular to the spine at the withers and over the 13th thoracic vertebra; at S3 the probe was placed cranial to the tuber sacralis, parallel to the spine; at S4 the probe was placed anterior to the tail-head at the level of the 1st to 4th coccygeal vertebrae, parallel to the spine; for S5 and S6 the probe was placed at the thoracic cage, over the 6th and 7th, and 12th and 13th intercostal spaces, respectively.

RTU images for SF thickness evaluation were obtained for 6 measurement sites described as follows [11]: site 1 (S1), the probe was placed perpendicular to the spine, at the level of wither (7th and 8th thoracic vertebra); site 2 (S2), the probe was placed perpendicular to the spine, at the level of the 13th thoracic vertebra; site 3 (S3), the probe was placed cranial to the *tuber sacralis* and 4 cm laterally and parallel to the spine; site 4 (S4), the probe was placed anterior to the tail-head at the level of the 1st to 4th coccygeal vertebrae, parallel to the spine; site 5 (S5), the probe was placed at the thoracic cage perpendicular to the 6th and 7th ribs, caudal to the point of the elbow; site 6 (S6), the probe was placed at the thoracic cage perpendicular to the 12th and 13th ribs, midway between the dorsal and ventral midlines (Figure 1). During RTU examination, the scanning sequence followed the same order from S1 to S6, for all the animals. All the images were taken on the left side, assuming small variation errors [23]; for each site, at least one good-quality image was recorded.

**Figure 2:**
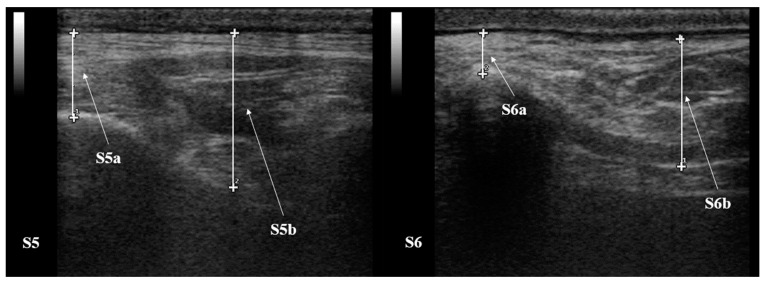
RTU image of S5 and S6 from the same healthy jenny at T5. SF was measured over the 6th rib (S5a) (white line number 1, in the panel on the left) and between ribs 6–7 (S5b) (white line number 2, in the panel on the left); at S6, the SF was measured over the 13th rib (S6a) (white line number 2, in the panel on the right) and between ribs 12–13 (S6b) (white line number 1, in the panel on the right). The pleura represents the lower limit of measurements. None of the measurements included the skin thickness. The left side of the images is cranial, the right side is caudal. B-mode, linear probe 7.5 MHz.

Measurements were performed offline by the same operator (IN), using a dedicated software (MyLab Desk, Esaote, Florence, Italy). For all the images obtained, the SF thickness was determined from subcutaneous fascia to proximal muscle fascia [11,24]. In particular, at S5, the SF was measured over the 6th rib (referred to on ultrasound image measurements as S5a) and between ribs 6–7 (referred to on ultrasound image measurement as S5b) (Figure 2); at S6, the SF was measured over the 13th rib (referred on ultrasound image measurements as S6a) and between ribs 12–13 (referred to on ultrasound image measurements as S6b), as shown in Figure 2. None of those measurements included skin thickness. For all SF evaluations, the average value of three consecutive measurements was considered, allowing us to overcome small variations of the SF, except for S5 and S6 [11].

### 2.5. Statistical Analysis

Data distribution was evaluated by Shapiro-Wilk analysis. Results concerning RTU SF thickness and BCS evaluated at each time point were reported as mean ± standard deviation.

An ANOVA test for repeated measures with Tukey’s test for multiple comparisons was applied to compare RTU SF measurements obtained for each RTU SF site (S1–S6) at each time (T1–T5).

A quantitatively complex relationship between RTU examinations (S1, S2, S3, S4, S5a, S5b, S6a, S6b) (independent variables) and BCS (dependent variable) was observed. A multiple linear regression (MLR) model was used to investigate the impact of several independent variables (X1, X2,…, Xk) on one dependent variable (Y). Multiple regression model takes the form:

Y = β0 + β1X1 + β2X2 +…+ βkXk + ε

where Y is the dependent variable; X1, X2,…, Xk are independent variables; β1, β2,…, βk are parameters; ε is a random component (the rest of the model).

MLR involved 6 jennies. Moreover, a residual analysis was performed to test if the regression model completely explained the studied association.

All the statistical analyses were performed with the Graph Pad Prism version 9 (Graph Pad Software, San Diego, CA, USA). Results were considered statistically significant with the value of *p* < 0.05.

## 3. Results

**Figure 3:**
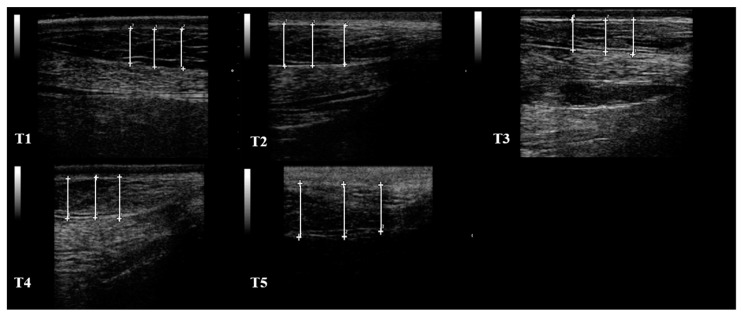
RTU image of S1 from the same healthy jenny, throughout the study period (T1, T2, T3, T4, T5). The SF thickness were determined from subcutaneous fascia to proximal muscle fascia and the average value of three consecutive measurements was considered (white lines), for each RTU image. The left side of the images is proximal, the right side is distal. B-mode, linear probe 7.5 MHz.

All the jennies enrolled in the present study met the inclusion criteria and showed no abnormalities trough the study period. Performing RTU was easy in field conditions, and the procedure was well tolerated by all the jennies. RTU visualization of SF was optimal in all the sites scanned. RTU images of S1 in one healthy jenny, throughout the study period, were reported in Figure 3.

A total of 180 images were evaluated. RTU measurement values of SF were normal distributed, thus results were reported as mean and standard deviation. RTU measurement values of SF for each site related to time were reported in Table 1.

**Figure 4:**
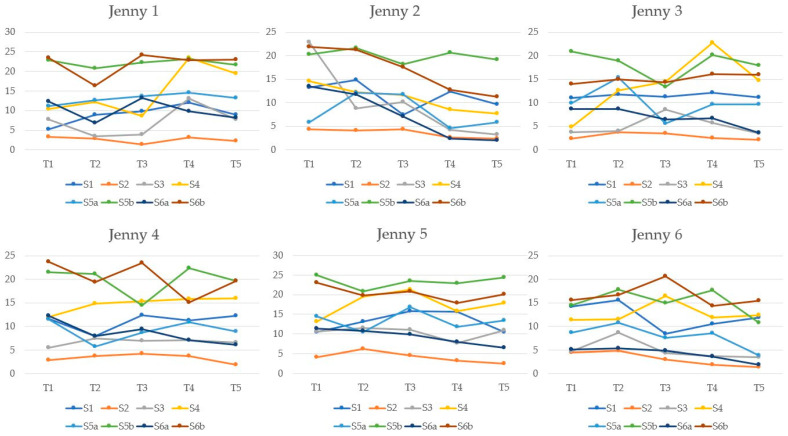
Individual jennies trends of RTU of SF, for each site (S1–S6b), throughout the study period (T1–T5). Legend: times (T1–T5) were reported on the x-axis, as follows: T1, 15 days before the presumptive parturition; T2, 7 days before the presumptive parturition; T3, 2 days after delivery; T4, 15 days after delivery; T5, 30 days after delivery. RTU of SF values was reported on the y-axis, in millimeters. Sites (S1–S6b) were reported as colored lines, as follows: S1, at the spine wither level; S2, at the spine, the 13th thoracic vertebra level; S3, cranial to the tuber sacralis, parallel to the spine; S4, anterior to the tail-head; S5 and S6, the probe was placed at the thoracic cage perpendicular to the ribs. In particular, for S5, the SF was measured over the 6th rib (referred to as S5a) and between ribs 6–7 (referred to as S5b), just caudal to the point of the elbow; at S6, the SF was measured over the 13th rib (referred to as S6a) and between ribs 12–13 (referred to as S6b).

Figure 4 shows individual animal trends of RTU of SF, through the study period related to observation time. Significant differences were found within RTU measurement values of SF for the following sites: S2 at T2 was different with respect to T5 (*p* = 0.004); S6a at T1 was different with respect to T5 (*p* = 0.02).

Prediction equations of BCS and linear effects of selected RTU examinations is reported in Table 2.

## 4. Discussion

Knowing the nutritional status and the potential changes in body energy reserve is essential in farm animals’ management, since these greatly affect animal productivity, health, and reproduction [11,25]. Body condition monitoring is an essential part of dairy cows’ herd management, aiming to continuously assess previous milk production and energy intake [14,15]. Similar to other dairy species, jennies show lower feed intake and an increase in NEFAs concentration during pregnancy and in the first months of lactation, confirming the difficulties in meeting the energy needs [25]. Various tools for estimating body energy reserve and balance have been studied through the years, such as respiration calorimetry estimation of body water content or measurement of fat cells size considered the gold standard [14,15]. However, these methods are not convenient for field practice. In the last decade, the backfat thickness evaluated by ultrasound has been chosen as an elective method in order to assess adipose storage tissue in dairy cows [14,15]. Since then, the ultrasound has been commonly used as an objective tool to measure the SF and tissue thickness; moreover, this has been successfully correlated to a correct assessment of the BCS in horses, donkeys and in food-producing animals [11].

To date, no information regarding donkeys’ reference RTU measurements values on deposition and distribution of fat in the different body regions was given [17]. Thus, our study could give preliminary indications on fat reserves in different body locations evaluated by using the RTU. Our experience showed that the RTU technique was easy to perform and well tolerated by the animals involved in the study representing a non-invasive and objective tool for body condition assessment in this species. RTU of SF could become an accepted method that can be performed easily and on large numbers of donkeys, as suggested in cows [26]. As reported previously [14], assessing backfat thickness in dairy cows resulted in easy to learn and quick to perform in the field, reporting that about 100 cows might be properly evaluated in 30–60 min. In the present study, the time required for performing RTU assessment in donkeys was not recorded, however, this might be a feature that needs to be investigated in further study, to assess the efficacy and effectiveness of the RTU technique.

The mean values of RTU measurements of SF from different sites during the whole study period were above those reported by Quaresma and colleagues (2013) [11], except for S5a and 5b which were similar. This might relate to the differences between the breeds used and to the different metabolism of jennies during the transition period compared to non-pregnant jennies evaluated in the study by Quaresma and colleagues [11]. Pregnant jennies bred for milking production seem to be characterized by major physiological challenges related to energy balance and fat mobilization, as shown in cows [27].

No significant difference has been detected during the observation of different sites through time, except for S2 and S6a: S2 at T2 and S6a at T1 were significatively different to values obtained at T5. For cows a significant effect of the time on the mean values of backfat thickness during the peripartum period was detected [27]: the values decreased gradually from 30 days before until 60 days after calving, as the peak of lactation approaches [27]. In jennies, the lactation curve is variable from 8 to 12 weeks post-partum. It usually presented 2 peaks, depending on the time of parturition: at 60 and 150 d in the summer group and a peak at 90 d in the spring group [28]. Approaching the peak of lactation might positively influence fat mobilization, since the first months of lactation led to difficulty in meeting the greater energy needs similarly to other dairy species [25,27]. In our study, differences were detected only in two sites probably because these are more susceptible to fat mobilization compared to others [12,13], which could be influenced since the earliest phase of lactation.

Results related to the association between BCS and sites through observational time have shown a good and reliable regression coefficient, with values of *p* = 0.001 and R^2^ = 0.52. As reported by others, RTU has been widely used to measure subcutaneous fat. It has been shown to be an objective tool to assess the body condition, to identify the deposit and distribution of fat in different body regions, in farm species and equids [11,14,17,29]. RTU technique has been shown a good sensitivity however weaknesses might be represented by technician ability, probe frequency and image capture and analysis [11,29], which might be affected also our results.

The present study showed some limits, such as the number of animals included was not high, which prevented us from setting reference values. In addition, comparing our study with research performed in cows, the study period was shorter, thus further works will need to increase the study population and to assess SF by RTU in jennies during a longer period of observation.

## 5. Conclusions

The present study could give preliminary indications on fat reserves in different body locations evaluated thanks to RTU, in pregnant and lactating jennies. RTU of SF measurements were above values reported for healthy donkeys, significative of different metabolic conditions of pregnant and lactating jennies; however, no variations were identified during the present study. RTU was a well-tolerated non-invasive technique, and it might represent an easy but also accurate tool for body condition assessment in donkeys during the transition period due to the correlation between this technique and BCS evaluation already shown by literature.

## Figures and Tables

**Table 1:** Mean and Standard deviation values for RTU measurements of SF for each site and BCS related to time. Data were reported in millimeters. Legend: T1, 15 days before the presumptive parturition; T2, 7 days before the presumptive parturition; T3, 2 days after delivery; T4, 15 days after delivery; T5, 30 days after delivery; S1, at the spine wither level; S2, at the spine, the 13th thoracic vertebra level; S3, cranial to the tuber sacralis, parallel to the spine; S4, anterior to the tail-head; S5 and S6, the probe was placed at the thoracic cage perpendicular to the ribs. In particular, for S5, the SF was measured over the 6th rib (referred S5a) and between ribs 6–7 (referred to as S5b), just caudal to the point of the elbow; at S6, the SF was measured over the 13th rib (referred as S6a) and between ribs 12–13 (referred as S6b); BCS: body condition score.

		T1	T2	T3	T4	T5
**S1** **(mm)**		10.95 ± 3.12	12.03 ± 3.09	10.87 ± 3.01	12.32 ± 1.75	10.72 ± 1.29
**S2** **(mm)**		3.60 ± 0.86	4.23 ± 1.18	3.50 ± 1.18	2.85 ± 0.64	2.12 ± 0.41
**S3** **(mm)**		9.17 ± 7.17	7.30 ± 3.10	7.47 ± 2.96	6.90 ± 3.37	5.92 ± 3.10
**S4** **(mm)**		11.70 ± 1.37	12.45 ± 1.23	14.90 ± 1.75	15.80 ± 2.40	15.35 ± 1.73
**S5** **(mm)**	**a**	10.25 ± 2.86	11.17 ± 3.19	10.70 ± 4.17	10.02 ± 3.37	9.17 ± 3.82
	**b**	20.83 ± 13.51	20.18 ± 1.49	17.78 ± 4.30	21.15 ± 2.10	18.92 ± 4.58
**S6** **(mm)**	**a**	10.55 ± 3.12	8.53 ± 2.38	8.47 ± 3.00	6.25 ± 2.76	4.72 ± 2.60
	**b**	20.28 ± 4.32	18.10 ± 2.42	20.17 ± 3.70	16.50 ± 3.56	17.58 ± 4.15
**BCS**		6.5 ± 0.63	6.33 ± 0.98	6.08 ± 1.39	6.17 ± 1.32	5.67 ± 1.29

**Table 2:** Prediction equations of body condition score and linear effects of selected RTU examinations evaluated in 6 jennies. S1, at the spine wither level; S2, at the spine, the 13th thoracic vertebra level; S3, cranial to the tuber sacralis, parallel to the spine; S4, anterior to the tail-head; S5 and S6, the probe was placed at the thoracic cage perpendicular to the ribs.

Prediction Equations	Constant	S1	S2	S3	S4	S5a	S5b	S6a	S6b	*p*-Value	Ac. R2%
**Y = a + b1X1 + b2X2 + b3X3 + b4X4 + b5X5 + b6X6 + b7X7 + b8X8**	3.00	0.03	−0.05	−0.10	0.12	0.02	0.10	0.30	−0.13	0.01	0.52

## Data Availability

The data presented in this study are available on request from the corresponding author.
